# Development of a sensitive primer extension method for direct detection and quantification of miRNAs from plants

**DOI:** 10.1371/journal.pone.0230251

**Published:** 2020-03-12

**Authors:** Ulku Baykal

**Affiliations:** Department of Genetics and Bioengineering, Giresun University, Güre, Giresun, Turkey; Kunming University of Science and Technology, CHINA

## Abstract

MicroRNAs (miRNAs) are short non-coding RNA molecules that regulate target gene expression in various organisms. Functional studies are therefore required to determine their temporal and spatial expression patterns. Primer extension has been used as a sensitive and reliable approach to identify miRNAs (∼21–22 nt) in the mammalian system and can be used in other systems such as plants. However, a well-defined method is required for ease of application and reproducibility. Here, a radioactive primer extension method was developed for the quantitative detection of miRNAs found in total RNA samples from plants. As a proof of concept, miR173 and miR828 were detected by primer extension in total RNA samples isolated from *Arabidopsis*. The assay involved the extension reaction of the miRNA guide strand with a radiolabeled specific primer. Using a manual DNA sequencer, primers extended with reverse transcriptase were separated on a denaturing polyacrylamide gel. The gel was then dried and exposed to a PhosphorImager screen for size-dependent product identification up to a single base difference. Quantification was done based on the intensity of radioactive signals by normalizing the cDNA products to an internal control. The primer extension was proven to be efficient to detect and quantify miRNAs in plant total RNA samples without subsequent enrichment of low-molecular-weight RNA species. This method, optimized for *Arabidopsis*, can be applied to a wide variety of organisms for the detection and quantification of miRNAs as well as siRNAs.

## Introduction

Small regulatory RNAs (sRNA) have been discovered in a variety of organisms, from plants to humans and even viruses. The biological processes they affect are growth and development, transcriptional and post-transcriptional gene silencing, chromosomal dynamics, and host defense [1, 2, 3]. Small RNA pathways have appeared in plants as defense mechanisms against RNA viruses and transposable elements and later evolved to regulate the expression of endogenous genes [4]. MicroRNAs (miRNAs) and small interfering RNAs (siRNAs) are the two major forms of small RNAs in plants. They are generated as single-stranded RNA molecules, approximately 18–25 nt-long, from the processing of different forms of double-stranded RNA (dsRNA) precursors. MicroRNAs are the most extensively studied class of small RNAs. They are key gene expression regulators, which involve in development, defense against invading pathogens and adaptation to abiotic stress, in plants by targeting the mRNA of protein-encoding genes for cleavage or translational repression. This complexity in the modes of action of miRNAs requires reliable detection and quantification of their expression for a better understanding of miRNA- mediated gene regulation.

Although miRNAs represent a relatively abundant class of transcripts, their expression levels vary greatly among different cells and tissues. Conventional technologies such as cloning, Northern hybridization, and microarray analysis are widely used, but may not be sensitive enough to detect less abundant miRNAs. Furthermore, intensive sequencing studies of small RNAs have revealed a very complex small RNA population in plants. Unlike mammals, which have relatively simple small RNA populations that mainly contain miRNAs and no siRNAs [5], plants have a rather complex small RNA fraction. It consists of both miRNAs and endogenous siRNAs derived from repetitive sequences, intergenic regions and genes [6, 7]. This diversity in the small RNA fraction leads miRNAs to be underrepresented and further affects the detection methods.

Several detection methods have been reported in the literature for profiling miRNAs, in addition to Northern blots [8, 9]. Many of these methods use hybridization between the target miRNA and a complementary strand nucleic acid probe [10]. Primer extension [11], Invader Assay [12], signal-amplifying ribozymes [13], splinted ligation [14], mirMASA bead-based technologies [15], quantitative reverse transcription-polymerase chain reaction (qRT-PCR) [16, 17], microarray [18, 19], and high-throughput sequencing [20] are among them. Northern blot is the oldest and most efficient standard approach for determining the size of a miRNA (both mature and its precursor [21, 22]) and also provides information on the expression level and validates the predicted miRNAs [23]. However, there are some disadvantages, such as the requirement for a large quantity and a high quality total RNA [24, 25].

Zeng and Cullen [11] have demonstrated that primer extension is a feasible method for quantitative analysis of miRNA expression in transiently transfected human cells. The initial use of primer extension is to identify transcriptional starting points. It is then widely used for validation of cloned small RNAs as well as for the quantitative analysis of small RNAs in tissues and cells. However, it has been found difficult to implement [26]. Another study has effectively applied primer extension for the analysis of miRNA and siRNAs in atasiRNA-mediated gene silencing in transgenic *Arabidopsis* plants [27]. However, there is no detailed report describing the method.

Here a sensitive primer extension method was developed to detect and quantify plant miRNAs. The potential of this approach was demonstrated by detecting the levels of miR173 and miR828 from *Arabidopsis* total RNA samples. The primer extensions of miR173 and miR828 were achieved by reverse transcription using radiolabeled specific primers. Then the resulting cDNA fragments were run on a denaturing polyacrylamide gel. The detection and the relative quantification were performed on the dried gel using a PhosphorImager.

## Results

### Assay optimization for quantitative detection of miRNAs

**Fig 1:**
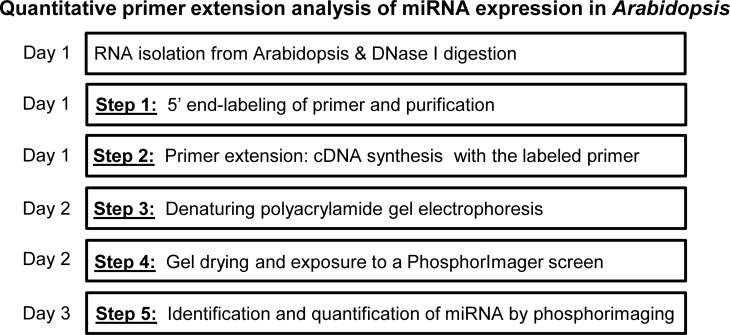
The workflow of the primer extension method for the detection and quantification of plant miRNAs.

To develop a quantitative primer extension assay for the analysis of plant miRNAs, some modifications were made to improve sensitivity and ease of use (see material and methods). This primer extension was a five-step assay: (1) The sequence-specific primers (miR173 and miR828 sequence from the database: PMRD [28]) were 5′ end-labeled with [γ-^32^P]ATP. (2) The radiolabeled primers were employed in the synthesis of cDNAs of mature miR173 and miR828 templates. No enrichment of low molecular weight RNA was performed for the miRNA primer extension protocol. (3) Synthesized cDNAs were run on a conventional polyacrylamide sequencing gel under denaturing conditions. (4) The gel used to separate cDNA products was dried to facilitate handling. (5) The dried gel was subjected to autoradiography and the results were analyzed using a PhosphorImager (Fig 1).

The percentage of denaturing polyacrylamide gel was optimized to 10% for the assay. This percentage was suitable both for separating cDNA fragments to distinguish a difference of 1 nt in molecular weight and for easy handling of the gel (without breaking during the drying process).

### Expression profiles of miR173 and miR828 in *Arabidopsis* plants

**Fig 2:**
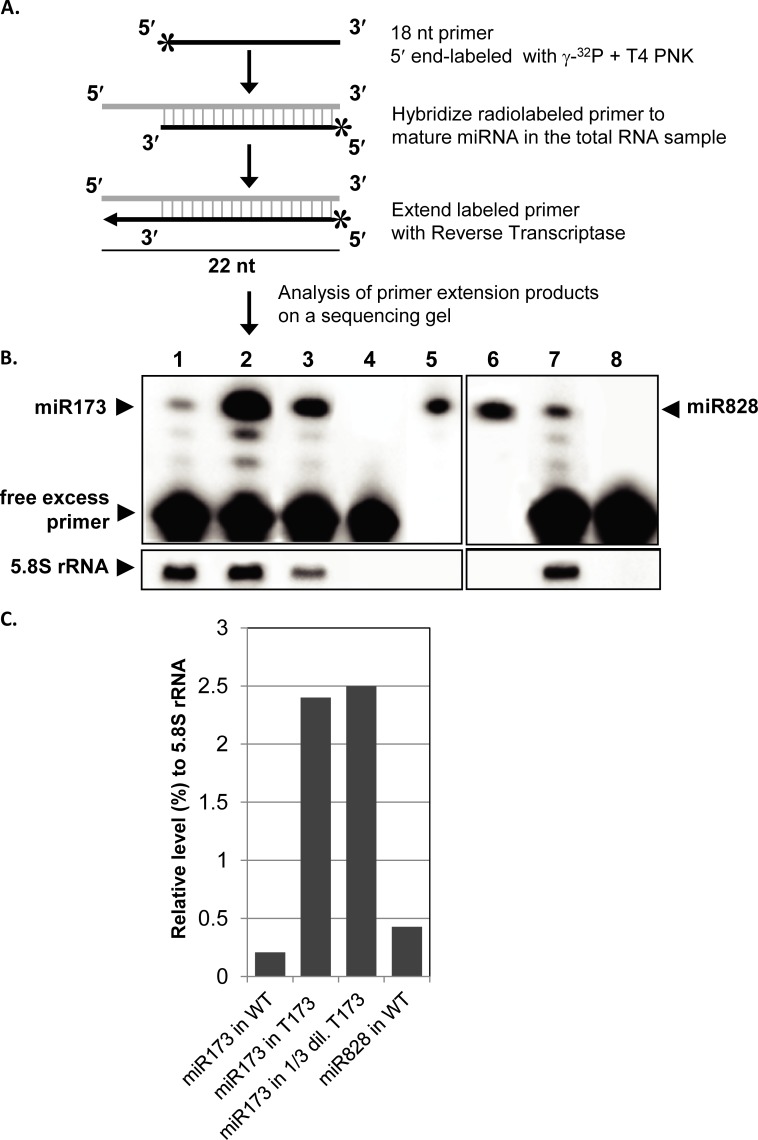
A primer extension method was developed and used to detect and quantify miR173 and miR828 expression in wild type and transgenic *Arabidopsis* plants. **(A)** A schematic representation of the primer labeling. The extension primers of *Arabidopsis* miR173 and miR828 were radiolabeled. **(B)** Primer extension assay to detect miR173 and miR828 in wild type *Arabidopsis* and miR173 in a transgenic *Arabidopsis* line (T173), which overexpresses miR173 from the CaMV35S promoter. Lane 1, miR173 in total RNA from the wild type *Arabidopsis*; lane 2, miR173 in total RNA from the *Arabidopsis* transgenic line, T173; lane 3, miR173 in the 1/3 diluted total RNA sample, which was used in lane 2; lane 4, [γ-^32^P]ATP-labeled specific primer used in the extension of miR173; lane 5, 22 nt, [γ-^32^P]ATP-labeled synthetic miR173 oligo; lane 6, 22 nt, [γ-^32^P]ATP-labeled synthetic miR828 oligo; lane 7, miR828 in total RNA from the wild type *Arabidopsis*; lane 8, [γ-^32^P]ATP-labeled specific primer used in the extension of miR828. 4 μg total RNA were used for each reaction and the loaded extension reaction contains ~1 μg of total RNA. The free probe is shown as a negative control for each miRNA analyzed. The primer extension products of miR173, miR828 or 5.8S rRNA were resolved on a 10% polyacrylamide/7 M urea gel and after drying exposed to a PhosphorImager screen overnight. **(C)** The percent miRNA expression was calculated by forming a ratio between the intensity of the expressed miRNA band (either miR173 or miR828) to the intensity of the 5.8S rRNA band and was plotted.

The primer extension method described here is for quantitative analysis of miRNA expression levels. The method was tested for two miRNAs differentially expressed in wild type Arabidopsis and a transgenic *Arabidopsis* line, which is a constitutive expressor of miR173. To determine the expression level of miR173 and miR828, a 22 nt fragment complementary to each corresponding mature miRNA was generated by reverse transcription with a sequence-specific radiolabeled primer (Fig 2A). The sequence-specific miRNA primers (18 nt), were shorter than regular PCR primers and promoted highly efficient primer extension of the miRNA template. The extension products and the molecular size markers were separated by using a 10% polyacrylamide/urea denaturing gel. The gel was then dried in a gel drier and analyzed with a PhosphorImager. The size of the bands detected on the gel relative to an oligonucleotide size marker labeled at the 5′ end. The 3′ end of the cDNA coincides with the 5′ end of the mRNA. Thus, the size of the radiolabeled cDNAs represented the distance from the labeled 5′ end of the primer to the 5′ end of the miRNA (i.e., the 3′ end of the cDNA). Fig 2B showed the expression profiles of *Arabidopsis* miR173 and miR828. The radioactive intensity of the extended specific probes showed a dose-dependent increase related to the complementary strand, the mature guide miRNA, found in the total RNA samples. Transgenic plants accumulated miR173 much higher than the wild type and miR828 was twice as high as miR173 in the RNA samples from wild type plants.

### Quantification of extended miRNA products

*Arabidopsis* miRNAs were detected and quantified using a quantitative primer extension in wild-type (miR173 and miR828) and transgenic *Arabidopsis* plants (miR173). Initially, *Arabidopsis* total RNA samples were reverse-transcribed using radiolabeled specific primers. Then, the reaction mixture containing the synthesized cDNAs were run on a denaturing polyacrylamide gel. Next, the gel was dried and exposed to a PhosphorImager screen. Afterward, the screen was scanned on a PhosphorImager for quantification. Sample-to-sample variation of miR173 and miR828 expressions in *Arabidopsis* total RNA samples was corrected by normalization with 5.8S ribosomal RNA (5.8S rRNA), which was chosen as a non-coding RNA (ncRNA) control. Normalization was performed by calculating miRNAs: 5.8S rRNA expression ratios. The primer extension of miR173 and miR828 revealed a significant difference in their expression levels in wild type plant. The amount of miR828 expression was more than twice as high as that of miR173. The constitutive expression of miR173 in the transgenic line, T173, was demonstrated to be much higher than that of the wild type plants. In addition to visual assessment (Fig 2B), their quantified expression levels (Fig 2C) also confirmed the differential expressions. Since the expression of miR173 is strictly regulated in wild type plants, its constitutive expression in the transgenics resulted in a much higher level than the wild type *Arabidopsis*. These results strongly suggest that primer extension is a powerful method to validate and quantify the expression of small RNAs in plants.

## Discussion

As an alternative to Northern blotting, a sensitive miRNA primer extension method was developed to detect and quantify mature miRNAs in plant RNA samples. MicroRNA validation method is usually Northern blotting. Limitations, such as the requirement of a high amount of total RNA, make it difficult to use in some situations. Despite improvements in sensitivity using LNA probes [24], the requirement for high amounts of RNA could not be reduced. UV cross-linking can also be challenging in Northern blotting due to the low sensitivity and reproducibility [29]. Therefore, primer extension can be used to validate miRNAs after microarray and small RNA sequencing.

Working with RNA is always difficult due to the presence of very robust and ubiquitous RNases. The primer extension eliminates the risk of RNA degradation during the detection process by converting miRNAs into cDNAs of the same size. The use of cDNA for detection shows that the assay is direct and does not require amplification of the target sequence. Therefore, the efficiency of the reaction observed and measured on the gel is directly proportional to the abundance of the target miRNA. Another important point in miRNA primer extension is the size of the primer used to detect miRNA. The primer can be shorter up to 2 nucleotides than the miRNA to be analyzed for better separation of the free probe. The difficulty in finding a primer that works well for a new target could be a disadvantage of primer extension. This can lead to problems with reverse transcription of certain miRNAs, however, such problems have not yet been reported. The assay specifically detected the miRNA of interest in total RNA extracts without a small RNA enrichment. The new generation reverse transcription enzymes allow extension reactions to be carried out at elevated temperatures by alleviating the problems associated with RNA secondary structures and increase the specificity of the gene-specific primer used in reactions. The primer extension method also reduces the risk of radioactive contamination by preventing laborious hybridization and washing steps of the Northern blot.

This robust and reliable primer extension method is important because it would help widen the scope of future plant small RNA research. Direct detection and quantification of miRNAs were accomplished without chemical or enzymatic modification of the target molecules. The entire protocol (from reverse transcription to the final analysis of results) was completed within three working days. Besides being more sensitive and higher throughput compared to the commonly used Northern blot, the method had the advantage of analyzing small RNAs from inadequate samples and could detect even a single base size difference. It is known that the expression levels of individual miRNAs affect their targeting properties [30]. The method can be used to analyze different expression levels of a miRNA in different samples to investigate its biological role. The anticipation is that this method would find use in the monitoring temporal, spatial, and disease patterns of the individual miRNA expression in plant tissues, and samples derived from experimentally tractable organisms. Furthermore, the method can also be used in the analysis of engineered siRNAs for targeted gene silencing.

## Materials and methods

### Day 1

#### Preparation of *Arabidopsis* total RNA

Wild type and transgenic (constitutive miR173 expresser) *Arabidopsis thaliana* seeds were germinated and grown *in vitro* on a sterile medium containing Murashige-Skoog salts [31] and 2% sucrose for 4 weeks under 16-hour long day conditions. The leaves were harvested from 4-week old plants and snap-frozen. Total RNA was isolated using the TRIzol (Invitrogen) according to the manufacturer’s instructions. Before cDNA synthesis, total RNAs were treated with 1 U/μl amplification grade DNase I (Invitrogen) to remove DNA contaminations. RNA concentration was quantified using a NanoDrop ND-1000 Spectrophotometer (NanoDrop Technologies, Wilmington, DE).

#### Oligonucleotide radiolabeling

Antisense oligos for the primer extension of miR173 and miR828 were designed based on the miRNA sequence registered in the miRBase Sequence Database. They were purchased from Integrated DNA Technologies (IDT). The 5′end-labeling of oligos was performed with [γ-^32^P]ATP using T4 polynucleotide kinase (Invitrogen). The sequences were as follows: At-miR173 extension primer 5′-GTGATTTCTCTCTGCAAG-3′, At-miR828 extension primer 5′-TGGAATACTCATTTAAGC-3′, 22 nt size markers: 5′-GTGATTTCTCTCTGCAAGCGAA-3′ and 5′-TCTTGCTTAAATGAGTATTCCA-3′, 5.8S rRNA (accession number: AT3G41979) extension primer: 5′-GTTCTTCATCGATGCGAGAGCCGAG -3.

The oligonucleotides were labeled at 5′ends by incubating 20 pmol oligonucleotide, 3 μl [γ-^32^P]ATP (10 μCi/μl, 3000 Ci/mmol) (Perkin-Elmer), 2 μl 10X forward reaction buffer, and 10 units T4 polynucleotide kinase (Thermo-Fisher) in a reaction volume of 20 μL for 10 min at 37°C. The reaction was stopped by heat inactivation (2 min at 90°C). The volume of the labeled products was adjusted to 50 μL with RNase-free water. The unincorporated radioactive labels were removed from radiolabeled oligonucleotides on a microspin G-25 column (GE Healthcare).

#### Primer extension of miRNA

The primer extension analysis of miR173 and miR828 was carried out essentially as described in the reports [32, 33]. A 5′end-labeled antisense primer and a reverse transcription kit, SuperScript III (Invitrogen), were used in the primer extension reaction. For reverse transcription, 8 μL of annealing master mix [4 μL DNase-treated total RNA (4 μg), 1 μL [γ-^32^P]ATP-labeled specific primer, 2 μL of DEPC-treated ddH_2_O, 1 μl of dNTPs (Invitrogen)] was heated to 65°C for 5 min and quickly chilled on ice for 2 min. The tube contents were collected by brief centrifugation. Then, 6 μL of buffer mixture [2.8 μL 5X first strand buffer, 1.5 μL 0.1 M DDT, 0.95 μL DEPC-treated water, 0.75 μL RNaseout (40 U/μL)] was added to the annealing mixture. Finally, an enzyme mixture for reverse transcription was prepared as follows: 3 μL of 5X first strand buffer, 11 μL of DEPC-treated water, 1 μL of SuperScriptIII Reverse Transcriptase (Invitrogen) and then 1 μL of the enzyme mixture was added to each primer extension reaction. The reaction was incubated at 50°C for 10 min and stopped by adding 10 μL of formamide loading dye (95% v/v deionized formamide, 10 mM EDTA (pH 8.0), 0.1% w/v xylene blue, 0.1% w/v bromophenol blue). For accurate relative quantification of miRNAs, the labeled 5.8S rRNA control primer was diluted 1:4 with the unlabeled 5.8S rRNA control primer before the primer extension of the 5.8S rRNA 5′ end fragment.

### Day 2

#### Denaturing polyacrylamide gel electrophoresis

A 10% denaturing polyacrylamide gel for miRNA detection was prepared as described in Theophilus with some modifications [34]: 20 ml of polyacrylamide from 30% acrylamide and bis-acrylamide solution (29:1), 25.2 gr urea, 6 ml of 10X TBE (Tris-Borate-EDTA) buffer [1 L, 10x TBE: 108 g of Tris base, 55 g of boric acid and 40 ml of 0.5 M Na_2_EDTA (pH 8.0) (Sigma-Aldrich)] were mixed and then the volume was completed to 60 ml with double distilled water. The solution was filtered to remove impurities. Electrophoresis plates were cleaned thoroughly with detergent followed by ethanol:water rinse. One of the plates was coated with dimethyldichlorosilane (GE Healthcare) and marked to identify the silanized side. The gel solution was prepared by adding and mixing 300 μL of 10% fresh ammonium persulfate (APS) (Bio-Rad) and 40 μL of TEMED (Bio-Rad) immediately to the 10% denaturing polyacrylamide gel solution at room temperature. The gel was allowed to polymerize for at least 1 h. A conventional sequencing gel apparatus (BRL) was used for casting with 30 x 40 cm glass plates and 0.4 mm, 25 tooth vinyl shark tooth combs and 0.4 mm side spacers. The gel was pre-run at 50 watts for 30 min before loading the sample. The primer extension reaction mixture (total 25 μL together with the loading dye) was denatured at 80°C for 10 min and then immediately placed on ice. Before loading the samples, urea was removed from the wells using a running buffer filled-10 ml syringe with a needle. After brief centrifugation, 6 μL of the samples (~1 μg of total RNA-containing extension reaction/lane) were loaded onto 7 M urea/ 10% polyacrylamide gel. Gel loading was done by leaving an empty lane between the samples. To obtain straight lines, the empty lanes were filled with the same concentration and amount of loading dye and run at 50 watts until the bromophenol blue dye reached ¾ of the gel.

#### Gel drying

After electrophoresis, one of the glass plates (silanized plate) was removed. 3MM filter paper, which has strong adhesion to sequencing gels, was used as a drying support to lift the gel from the glass plate. The gel on the filter paper was covered with a Saran wrap film. Then, the gel was dried in a slab gel dryer (Bio-RadModel 583 Gel Dryers) at 80°C for 1 h (until the gel was dry).

### Day 3

#### Gel imaging

The dried gel was exposed to a PhosphorImager screen overnight to scan. Radioactive bands were visualized and the amount of miRNA in the primer extension gels was quantified relatively using the Phosphoimager (MolecularDynamics).
